# Lexical and Phonetic Influences on the Phonolexical Encoding of Difficult Second-Language Contrasts: Insights From Nonword Rejection

**DOI:** 10.3389/fpsyg.2021.659852

**Published:** 2021-05-31

**Authors:** Miquel Llompart

**Affiliations:** Chair of Language and Cognition, Department of English and American Studies, Friedrich Alexander University Erlangen-Nuremberg, Erlangen, Germany

**Keywords:** second language learning, lexical representation, speech perception, L2 lexicon, phonolexical encoding, lexical decision, nonword rejection, L1-accented input

## Abstract

Establishing phonologically robust lexical representations in a second language (L2) is challenging, and even more so for words containing phones in phonological contrasts that are not part of the native language. This study presents a series of additional analyses of lexical decision data assessing the phonolexical encoding of English /ε/ and /æ/ by German learners of English (/æ/ does not exist in German) in order to examine the influence of lexical frequency, phonological neighborhood density and the acoustics of the particular vowels on learners’ ability to reject nonwords differing from real words in the confusable L2 phones only (e.g., *l[æ]mon, *dr[ε]gon). Results showed that both the lexical properties of the target items and the acoustics of the critical vowels affected nonword rejection, albeit differently for items with /æ/ → [ε] and /ε/ → [æ] mispronunciations: For the former, lower lexical frequencies and higher neighborhood densities led to more accurate performance. For the latter, it was only the acoustics of the vowel (i.e., how distinctly [æ]-like the mispronunciation was) that had a significant impact on learners’ accuracy. This suggests that the encoding of /ε/ and /æ/ may not only be asymmetric in that /ε/ is generally more robustly represented in the lexicon than /æ/, as previously reported, but also in the way in which this encoding takes place. Mainly, the encoding of /æ/ appears to be more dependent on the characteristics of the L2 vocabulary and on one’s experience with the L2 than that of its more dominant counterpart (/ε/).

## Introduction

A crucial part of second language (L2) learning is building a non-native lexicon. This can be a very challenging endeavor, especially when the L2 is learned later in life and in a non-immersion setting, as is the case for many learners of English around the world (e.g., Díaz et al., 2012). For this type of learners, much of the learning takes place in a formal instruction setting (i.e., classroom) and the L2 is rarely spoken outside of that environment. This rather constrained interaction with the L2 has apparent negative consequences on the acquisition of non-native lexical items. First, the relatively impoverished input translates into reduced exposure to individual L2 words, which often prevents their robust integration into long-term memory (Gollan et al., 2008) and almost invariably results in smaller vocabulary sizes in the L2 when compared with the native language (L1; Nation, 2006). Secondly, for words that become part of the L2 lexicon, the scarcity of L2 input results in the newly established lexical representations being phonologically vague or “fuzzy” (Cook and Gor, 2015; Cook et al., 2016; Lancaster and Gor, 2016). This means that the encoding of phonetic categories into lexical representations (i.e., phonolexical encoding) is not as robust as that of native lexical items, which greatly contributes to L2 spoken word recognition being rather error-prone and characterized by spurious lexical competition (e.g., Weber and Cutler, 2004; Cook et al., 2016).

An additional obstacle for the establishment of robust L2 lexical representations is that learners are bound to face difficulties while trying to master the phonology of the non-native language. In particular, L2 phonological contrasts that are not part of the L1 are very often the source of perceptual difficulties. This is the case, for example, of the English distinction between /r/ and /l/ for native speakers of Japanese (Goto, 1971; Bradlow et al., 1999) and the vowel contrast between /ε/ and /æ/ for L1-German learners of English (Llompart and Reinisch, 2017, 2019a, 2020; Eger and Reinisch, 2019a,b), which is the object of the present study. Both /r/-/l/ and /ε/-/æ/ are instances of what Best and Tyler (2007) labeled as single-category assimilations in their model of L2 phonology learning; that is, a scenario in which two L2 phones are perceived as being perceptually close to one and the same L1 phone. It has been repeatedly shown that perceptual difficulties with L2 contrasts in single-category-assimilation relationships lead to representational imprecisions for words containing these contrasts (e.g., Broersma, 2012; Llompart and Reinisch, 2019b). Importantly, these imprecisions are long-lasting in that they appear to be in place even after L2 speakers have already learned to perceive the phonetic differences between the L2 phones (Díaz et al., 2012; Darcy et al., 2013; Amengual, 2016; Llompart, 2021). For example, Llompart (2021) provided evidence of a weak encoding of the /ε/-/æ/ contrast into English words even by German learners of English who had had extensive experience with the L2 and were able to distinguish between the two vowels in a phonetic identification task.

A task that has recurrently been used to assess the phonological robustness of lexical representations in late L2 learners is lexical decision involving real words and “mispronounced” nonwords. In such a task, words of the L2 are auditorily presented either in their canonical form or containing systematic phonological substitutions that transform them into nonwords. Participants are then asked to decide whether the items presented are real words in the L2 (Díaz et al., 2012; Darcy et al., 2013; Darcy and Thomas, 2019; Llompart and Reinisch, 2019b; Melnik and Peperkamp, 2019, 2021). Lexical decision tasks of this type have helped shed light on several issues concerning the phonolexical encoding of challenging L2 contrasts. First, lexical decision data have served to support the finding of previous visual-world eye tracking studies (Weber and Cutler, 2004; Cutler et al., 2006) that the encoding of these challenging contrasts is asymmetric and modulated by the goodness-of-fit of the L2 categories to the closest L1 category. As discussed by Cutler et al. (2006), the better-fitting L2 category in the contrast (i.e., more similar to the L1 category) is thought to be dominant and more robustly encoded into the corresponding L2 words than the worse-fitting alternative, whose encoding is generally less precise. For /ε/-/æ/, /ε/ is attributed this dominant role, to the point that /æ/ has been re-labeled in previous studies as not-/ε/ or */ε/ to emphasize its weaker phonolexical encoding (Llompart and Reinisch, 2017; see also Hayes-Harb and Masuda, 2008). Lexical decision data from Dutch (Simon et al., 2014) and German learners of English (Llompart and Reinisch, 2019b; Llompart, 2021) has contributed to characterizing this asymmetry by showing that learners are more sensitive to vowel substitutions when the target vowel should be /ε/ (e.g., *l[æ]mon) than in contexts in which it should be /æ/ (e.g., *dr[ε]gon). Secondly, recent research using this paradigm has examined the role that individual differences within the learner population may play with regard to phonolexical encoding. Here findings suggest that a more robust encoding of difficult L2 contrasts relates to learners’ phonetic categorization ability for that particular contrast (Silbert et al., 2015; Simonchyk and Darcy, 2017; Darcy and Holliday, 2019) as well as to their L2 vocabulary size (Daidone, 2020; Llompart, 2021).

What has not received much attention in this particular body of literature, however, is the role that item-specific properties, both lexical and phonetic, may play on learners’ ability to accept real words containing confusable L2 phones and successfully reject nonwords that differ from real words in those particular phones. This is the case even though an examination of such properties could be crucial for our understanding of the influence that lexical factors may have on the phonolexical encoding of phones in challenging L2 contrasts, an issue that is not well-understood yet. As a first step in this direction, the present study presents a series of additional analyses on lexical decision data from German learners of English (as reported in Llompart and Reinisch, 2019b, and Llompart, 2021) aimed at assessing the effects of L2 words’ lexical frequency, phonological neighborhood density and the acoustics of the critical vowel on learners’ ability to reject nonwords containing /ε/-/æ/ mispronunciations (e.g., *l[æ]mon, *dr[ε]gon). While the role that these factors may play with respect to accuracy in real word acceptance is also a question of theoretical interest, real word acceptance rates were not assessed in this study because of learners’ ceiling performances with real /ε/- and /æ/-words in Llompart and Reinisch (2019b) and Llompart (2021)^1^.

For responses to mispronounced nonwords, it is in principle expected that both the lexical properties of the items presented and the acoustic image of the auditory stimuli corresponding to these items should influence learners’ lexicality decisions. Concerning lexical frequency, nonwords built on high-frequency words could be expected to be harder to reject than nonwords based on lower-frequency words. Lexical decision tasks as the ones described above rely on the well-documented Ganong effect (Ganong, 1980) to bias participants’ responses toward considering the stimuli “real words.” Ganong (1980) created a stimulus that was ambiguous between /t/ and /d/, appended the same stimulus to *-ask* and *-ash* and asked listeners to categorize the initial phone as /t/ or /d/ in each context. Listeners were found to be more likely to categorize it as /t/ in the ?ask context and as /d/ in the ?ash context, thus showing that lexical knowledge guides speech perception when the signal is acoustically ambiguous. Following from this, in the present experimental paradigm, in which listeners are presented with items like *dr[ε]gon and asked whether they are real words of English or not, they are expected to be more likely to answer “word” than “nonword” whenever acoustic information is not enough for them to be certain of the identity of the substituted or mispronounced phone. Crucially, this attraction toward “word” responses should be stronger the more frequently listeners have encountered the words that served as a base form for the nonwords in the L2 (Coltheart et al., 1977; Andrews, 1996; Perea et al., 2005; but see Politzer-Ahles et al., 2020).

Like lexical frequency, phonological neighborhood density is also known to have a major impact on lexical access, and more specifically, on lexicality decisions. However, for the task examined here, it is unclear whether high phonological neighborhood densities should aid or prevent the accurate rejection of mispronounced nonwords. On the one hand, given that higher phonological neighborhood density tends to hinder auditory word recognition by enhancing lexical competition (Luce and Pisoni, 1998; Vitevitch, 2002a,b), higher densities could be expected to bias listeners toward “word” responses for the nonwords in a similar way as high lexical frequencies should. On the other hand, one could alternatively predict that higher neighborhood densities may boost accurate nonword rejection. Higher phonological neighborhood densities should almost invariably mean larger clusters of similar-sounding words containing the same L2 target phone. Hence, especially for difficult L2 phonological contrasts, the existence of multiple phonologically similar word forms with a specific L2 category in the contrast (and not the other) may be beneficial for the establishment of robust links between the corresponding phonetic category and L2 word forms (see Llompart, 2021). Because of this, a scenario in which accuracy in nonword rejection increases as a function of the number of neighboring words with the same target category a given form has is also plausible.

Regarding the acoustic properties of the relevant L2 phones in the nonwords, lexical decision tasks generally use stimuli in which the mispronunciations were elicited naturally, and this is the case in Llompart and Reinisch (2019b) and Llompart (2021), the studies that provided the dataset to be analyzed here. By design, the use of naturally elicited stimuli means that the target phones must show some variation in their acoustics, most likely related to the surrounding phones (e.g., Strange et al., 2007) and to inherent within-speaker variation. Hence, a relevant question here, and one which has not yet been addressed, is how sensitive learners are to fine-grained acoustic variation in a task where they are mainly asked to focus on the lexicality of the stimuli. In principle, one could predict that, for nonwords with systematic vowel substitutions, the more acoustically distinctive the substitution, and thus the further from the canonical vowel the “mispronounced” vowel is, the easier it should be for learners to detect the mismatch and reject these nonwords. While this is a likely possibility for mispronunciations involving L2 phones not leading to perceptual difficulties, it is less clear that this should be the case for nonwords containing perceptually confusable non-native phones like /ε/ and /æ/ for German learners of English. Since the phonetic categories for these phones are most likely not as well-defined, learners may fail to use between-item variation in the acoustics of the stimuli as a cue to their judgments (Díaz et al., 2012; Llompart and Reinisch, 2019a).

Finally, it is worth noting that, in Llompart and Reinisch (2019b) and Llompart (2021), there were two different types of mispronounced nonwords for the L2 contrast of interest (/ε/-/æ/). These were items in which /ε/ was substituted by [æ] (e.g., *l[æ]mon) and items in which /æ/ was substituted by [ε] (e.g., *dr[ε]gon). Crucially, these two types differ in two important respects. The first is the difference in goodness of fit to the closest L1 category of the canonical vowel (i.e., /ε/ > /æ/) and the mispronounced vowel realizations (i.e., [ε] > [æ]), which, as was discussed above, is bound to have consequences on the perception and lexical encoding of these phones. The second key difference is that, for learners in a non-immersion setting, the two mispronunciation types are not equally likely to conform to their experience in their day-to-day L2-learning environment. Germans learning English in Germany are extremely likely to be exposed to instances in which /æ/ is produced with acoustic properties more closely aligning with /ε/ (e.g., h[ε]ppy, pl[ε]n, dr[ε]gon) in the speech of fellow learners and perhaps even English teachers (see Eger and Reinisch, 2019a; Llompart and Reinisch, 2019a for acoustic data), whereas the opposite pattern (e.g., st[æ]p, l[æ]mon) is very unlikely to occur. Therefore, these critical disparities warrant the additional question as to whether the effects of lexical frequency, phonological neighborhood density and vowel acoustics could differ between the two types of mispronounced nonwords examined.

## Materials and Methods

### Participants

Data from 116 participants were included in all analyses presented in the Results section. Thirty-seven participants were the German learners of English (19 females, mean age = 25.32, *SD* = 4.37) included in the analyses of Llompart and Reinisch (2019b). These participants were students at the Ludwig Maximilian University of Munich (LMU) who had grown up in German monolingual households, had not spent more than 6 months in an English-speaking country and were not enrolled in courses administered by the English department of the university. The remaining 79 participants were the two groups of learners tested in Llompart (2021). The first group consisted of 49 German learners of English studying at the Friedrich Alexander University Erlangen-Nuremberg (FAU; 35 females, mean age = 24.22, *SD* = 4.26) who were recruited according to the same criteria as the previous group. The second group consisted of 30 English professionals and university students studying to become English professionals also recruited at FAU (17 females, mean age = 28.5, *SD* = 12.32). These were either language instructors at the university’s Language Center (*N* = 5) or students enrolled in the BA and MA programs offered by the Department of English and American Studies (*N* = 25). In the present study, and following Llompart (2021), the first two groups will be commonly referred to as “intermediate” German learners of English and the last group will be henceforth referred to as “advanced” German learners of English. Detailed information on self-reported proficiency and language use measures for these participants, elicited by means of language background questionnaires, can be found in Llompart and Reinisch (2019b) and Llompart (2021)^2^.

### Materials

All participants took part in the same lexical decision task. In this task, real words of English as well as nonwords created by applying systematic phonological substitutions to real words were presented auditorily and participants had to decide whether each stimulus was a real word of English or not. As described in Llompart and Reinisch (2019b) and Llompart (2021), materials included 304 English unique words, of which 52 contained the phones in the difficult L2 contrast /ε/-/æ/. The remaining 252 words involved 5 contrasts (/i/-/I/, /ɔ:/-/u/, /p/-/t/, /k/-/m/ and /b/-/v/) that were expected to be unproblematic for native German speakers. Importantly, half of the words were selected to appear in the task as canonically produced, while the other half was presented as nonwords in which the phones in the relevant contrasts were exchanged. Hence, the sets of canonically produced words and mispronounced nonwords contained different lexical items. For /ε/-/æ/ this meant that 13 words with /æ/ appeared with /æ/ produced as [æ] and 13 different words were presented with /æ/ mispronounced as [ε] (h[æ]mmer vs. *dr[ε]gon). The same manipulation held for items with /ε/ (d[ε]sert vs. *l[æ]mon), and the same procedure was also applied to filler contrasts. While for the critical items the target was always the first stressed vowel, for fillers the position of the critical phones in the word could vary. All 304 words were recorded by a male speaker of Southern British English in their correct form and half of the items, that is, those designed to appear as nonwords, were also recorded with the suitable phonological substitutions.

### Procedure

Participants were tested either in a sound-attenuated booth or a quiet room at their respective universities. The lexical decision task was implemented in Psychopy 2 (in Llompart and Reinisch, 2019b; v. 1.83.01) or Psychopy 3 software (in Llompart, 2021; v. 3.0.2; Peirce et al., 2019). Auditory stimuli were presented over headphones at a comfortable listening level. Before the start of the task, participants were instructed that they would listen to a native English speaker say English words and invented words that could in some cases sound similar to English words. Their task was to indicate, for each item, whether they considered it to be a real word in English. On each trial, two boxes were shown on the screen, a green one with “word” written on it on the left-hand side and a red one with “not a word” written on it on the right-hand side, and an auditory stimulus was presented. Participants had to press “1” on a numeric keyboard (in Llompart and Reinisch, 2019b) or the leftmost button of a response pad (in Llompart, 2021) to indicate that the auditory stimulus was a real word, and “0” or the rightmost key of the response pad if they considered that the stimulus was not a real word. There was no time limit for participants’ responses. The 304 items were presented in a randomized order. Before the start of the task, participants were presented with 10 practice trials in order to familiarize them with the procedure. It took participants approximately between 15 and 20 minutes to complete the task.

## Results

All analyses focused on /ε/- and /æ/-nonwords only, that is, the 13 items containing /ε/ → [æ] mispronunciations (e.g., *l[æ]mon) and the 13 items containing /æ/ → [ε] mispronunciations (e.g., *dr[ε]gon), respectively. Lexical frequencies and phonological neighborhood densities for these items were calculated in order to assess whether these lexical factors modulated participants’ responses to the nonwords in the lexical decision task. Lexical frequency was assessed through the Zipf-scale frequency measures of Subtlex-UK (van Heuven et al., 2014) and neighborhood density was determined by consulting CLEARPOND (Cross-Linguistic Easy-Access Resource for Phonological and Orthographic Neighborhood Densities; Marian et al., 2012). In addition, for the acoustic stimuli corresponding to these items, the F1 and F2 values (in hertz) of the critical vowels at vowel midpoint were extracted using a Praat script (Boersma and Weenink, 2009) so that the potential impact of the acoustics of the mispronounced vowel could also be examined. The difference score between F2 and F1 (F2–F1) was then calculated for each item in order to be able to use only one value per item in the analyses. In British English, [ε] is known to have a lower F1 and a higher F2 than [æ] (Deterding, 1997; Llompart and Reinisch, 2017). Therefore, the F2–F1 difference should always be higher for [ε] than [æ]. This was the case in the present stimuli, as the mean F2–F1 of the /æ/-nonwords, in which the first vowel was produced like [ε], was 1,187 Hz (*SD* = 91), and the mean F2–F1 of the /ε/-nonwords (i.e., with /ε/ produced like [æ]) was 568 Hz (*SD* = 103). The F2–F1 value of the critical vowel in each of the /ε/- and /æ/-nonwords is provided in Table 1, together with the lexical frequency and phonological neighborhood density of the word from which the nonword was derived. Correlational analyses over the set of 26 nonwords showed that lexical frequency and phonological neighborhood density were not correlated with each other [*r*(24) = 0.08, *p* = 0.69] and neither of them was significantly correlated with the F2–F1 values of the critical vowels either [lexical frequency: *r*(24) = −0.28, *p* = 0.17; phonological neighborhood density: *r*(24) = −0.23, *p* = 0.27].

**TABLE 1:** Lexical frequency, phonological neighborhood density, and F2–F1 values of the critical vowels for each of the /ε/- and /æ/-nonwords analyzed in the present study.

nonword item	Lexical frequency (Subtlex-UK)	Phonological neighborhood density (CLEARPOND)	Vowel acoustics (F2–F1 in hertz)
ch[æ]rry	4.23	11	710
ch[æ]ss	3.91	18	592
d[æ]sk	4.34	6	685
dr[æ]ss	4.85	9	621
fr[æ]sh	4.89	4	513
h[æ]lth	5.14	9	356
h[æ]lp	5.75	12	433
l[æ]gend	4.34	1	615
l[æ]mon	4.46	5	546
l[æ]sson	4.46	5	471
s[æ]ntence	4.34	1	592
w[æ]ther	5.12	12	578
y[æ]llow	4.84	10	679
ch[ε]nnel	4.51	6	1,086
dr[ε]gon	4.28	1	1,251
f[ε]ctor	4.5	7	1,189
f[ε]ctory	4.59	0	1,232
fl[ε]g	4.42	11	1,189
g[ε]llery	4.28	2	1,151
h[ε]bit	4.04	4	1,291
l[ε]mp	4.09	14	1,118
r[ε]mp	3.67	16	1,055
sc[ε]ndal	4.22	3	1,332
spl[ε]sh	4.13	3	1,045
st[ε]ndard	4.72	2	1,228
th[ε]nk	5.87	11	1,259

Prior to any analyses, lexical decision data corresponding to responses to /ε/- and /æ/-nonwords were first trimmed by excluding all trials that contained nonwords based on words with which participants were not familiar. This was assessed by means of a word familiarity questionnaire administered after the lexical decision task. Only 26 trials were excluded on these grounds (0.86% of all /ε/- and /æ/-nonword trials). Before directly assessing the influences of lexical frequency, phonological neighborhood density and vowel acoustics on learners’ responses, data were first submitted to a generalized mixed-effects regression model with a logistic linking function (lme4 package 1.1–23 in R version 3.6.3; Bates et al., 2015) on accuracy data with Vowel [/ε/ (produced as [æ]; *l[æ]mon) -/æ/ (produced as [ε]; *dr[ε]gon)] and Group (intermediate in Llompart and Reinisch (2019b), intermediate in Llompart (2021) and advanced in Llompart (2021)) as variables of interest. This model was devised to be used as the base model on which the effects of lexical frequency, phonological neighborhood density and vowel acoustics were to be subsequently tested (see below).

The base model had Response (0 = incorrect, 1 = correct) as categorical dependent variable. Vowel was contrast coded such that /ε/ was coded as −0.5 and /æ/ as 0.5. Group was re-coded as two linearly independent contrasts which will be henceforth referred to as “Proficiency” and “Study.” “Proficiency” was coded to capture differences in accuracy between the two groups of intermediate learners and the group of advanced learners. Hence, trials for the former two groups were coded with −0.25, and trials corresponding to the latter were coded as 0.5. “Study” was included to assess potential differences between the two intermediate groups of learners, who were recruited as part of two different studies and in two different institutions but following the same recruiting requirements. Data from the intermediate learners in Llompart and Reinisch (2019b) were coded as −0.5, data from the intermediate learners in Llompart (2021) were coded as 0.5, and data from the advanced participants in the same study were coded as 0. Proficiency and Study were not allowed to interact but the interactions between each of these predictors and Vowel were included. The random effects structure consisted of random intercepts for Participants and a random slope for Vowel over Participants. Random intercepts for Items were not included because Item co-varied with lexical frequency, phonological neighborhood density and vowel acoustics (i.e., each item had one value for each variable) and would thus be problematic for the additional analyses examining their effects.

The model revealed significant effects of Vowel (*b* = −0.98; z = −9.68; p < 0.001) and Proficiency (*b* = 1.58; *z* = 5.66; *p* < 0.001). The effect of Study was not significant (b = −0.03; z = −0.14; p = 0.89) and neither were the interactions between Vowel and Proficiency and Vowel and Study (both *p* > 0.1). Hence, listeners were found to be more accurate with /ε/ → [æ] mispronunciations (/ε/-nonwords, e.g., *l[æ]mon; *M* = 50.67% correct, *SD* = 50.01) than with /æ/ → [ε] substitutions (/æ/-nonwords, e.g.,*dr[ε]gon; *M* = 31.52% correct, *SD* = 46.47) across the board, and learners labeled as advanced in Llompart (2021) were more accurate overall (*M* = 58.61% correct, *SD* = 49.28) than the two groups of intermediate learners (2019: *M* = 35.5% correct, *SD* = 47.88; 2021: *M* = 34.6% correct, *SD* = 47.59), whose nonword rejection accuracies were almost identical.

After this, separately for each of the three predictors of interest (i.e., lexical frequency, phonological neighborhood density and vowel acoustics), forward stepwise model comparisons (Zhang et al., 2020) were conducted between i) the base model described above (random-effects structure: Vowel|Participant) and a model including random slopes for one of the predictors over Participants (e.g., Vowel + Frequency|Participant), and ii) between the model including the random slopes only and a model including these random slopes plus an interaction term with Vowel over Participants (e.g., Vowel*Frequency|Participant). Comparisons were conducted by means of log-likelihood tests using the anova() function in R. These comparisons assessed whether the additional complexity of the random-effects structure improved the models’ fit. In particular, the comparisons between the base model and the models with only random slopes were performed to ascertain whether lexical frequency, phonological neighborhood density and vowel acoustics modulated participants lexicality responses across the board, while the comparisons between the models with and without the interaction terms determined whether the effects were qualified by the type of nonword items (/ε/-nonwords vs. /æ/-nonwords).

This analytical procedure was selected because it allowed for the examination of item-specific effects in an independent way while still taking into account the population-level effects that had already been reported in the previous studies. By analyzing whether allowing the model to account for variation caused by participants’ diverging sensitivities to lexical frequency, neighborhood density and vowel acoustics improved the fit of the model to the actual data, it could be determined whether these properties of the individual items affected participants’ responses without having to deal with drawbacks that would have been unavoidable if these predictors had simply been added to the fixed-effects structure of the model. First, this approach avoided that the effects of item-specific properties were knowingly overestimated, as it would have been the case if they had been analyzed as the sole fixed effects, disregarding thus the effects that both target vowel and differences between learner groups have been shown to have in previous studies. Secondly, and very much relatedly, this procedure also prevented that the contributions of the item-specific measures investigated were obscured by the robust effects of the aforementioned variables.

Results of the model comparisons between the base model and three separate models including random slopes for Lexical Frequency, Phonological Neighborhood Density and Vowel Acoustics, respectively, over Participants showed that the addition of a random slope for Lexical Frequency over Participants improved the model’s fit [χ^2^(3) = 8.50, *p* < 0.05], and so did adding a slope for Vowel Acoustics [χ*^2^*(3) = 36.61, *p* < 0.001]. By contrast, adding a slope for Neighborhood Density did not result in an improvement [χ^2^(3) = 1.44, *p* = 0.70]. Furthermore, comparisons between the models with random slopes only and models including an interaction term with Vowel revealed that the interaction terms between Vowel and Lexical Frequency over Participants [χ^2^(4) = 17.52, *p* < 0.01] and between Vowel and Neighborhood Density [χ^2^(4) = 28.80, *p* < 0.001] improved the fit of the respective models. The model including an interaction between Vowel Acoustics and Vowel over Participants had severe convergence issues that rendered it uninterpretable. However, a comparison involving simplified models in which the non-significant interactions between Vowel and Proficiency and Vowel and Study were removed from the fixed-effects structure showed that adding an interaction term between Vowel Acoustics and Vowel over Participants to the random-effects structure considerably improved the fit of the simplified model with random slopes for Vowel Acoustics only [χ^2^(4) = 29.50, *p* < 0.001].

**FIGURE 1:**
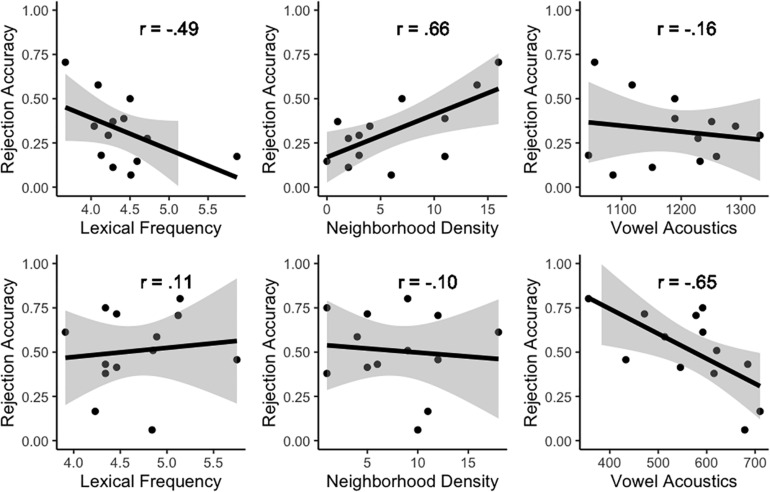
Scatterplots of accuracy in nonword rejection for /æ/-nonwords (top row) and /ε/-nonwords (bottom row) as a function of lexical frequency (left), neighborhood density (center), and vowel acoustics (right). Regression lines and correlation coefficients (i.e., *r*) are provided for illustration purposes.

Based on the significant improvements in model fit stemming from the addition of interaction terms to the random-effects structure, data were split by Vowel and the effects of adding random slopes for Lexical Frequency, Neighborhood Density and Vowel Acoustics were quantified for each vowel separately by comparing a base model with only random intercepts (1| Participant) to models with random slopes for Lexical Frequency, Phonological Neighborhood Density and Vowel Acoustics, respectively, over Participants (e.g., Frequency|Participant). For /æ/-nonwords (e.g., *dr[ε]gon), slopes for Lexical Frequency [χ^2^(3) = 21.88, *p* < 0.001] and Neighborhood Density [χ^2^(3) = 26.07, *p* < 0.001] over Participants improved the model’s fit, while a slope for Vowel Acoustics did not [χ^2^(3) = 1.00, *p* = 0.80]. For /ε/-nonwords (e.g., *l[æ]mon), the opposite pattern emerged. A random slope for Vowel Acoustics over Participants substantially improved the model’s fit [χ^2^(3) = 63.29, *p* < 0.001] while slopes for Lexical Frequency [χ^2^(3) = 0.40, *p* = 0.94] and Neighborhood Density [χ^2^(3) = 1.75, *p* = 0.62] did not do so. These results align perfectly with the patterns observed in the raw data presented in Figure 1, which provides scatterplots of accuracy in nonword rejection for /æ/-nonwords (top row) and /ε/-nonwords (bottom row) as a function of lexical frequency (left), neighborhood density (center) and vowel acoustics (right). Regression lines and correlation coefficients (i.e., *r*) are also provided to better outline the relationships between these variables.

**FIGURE 2:**
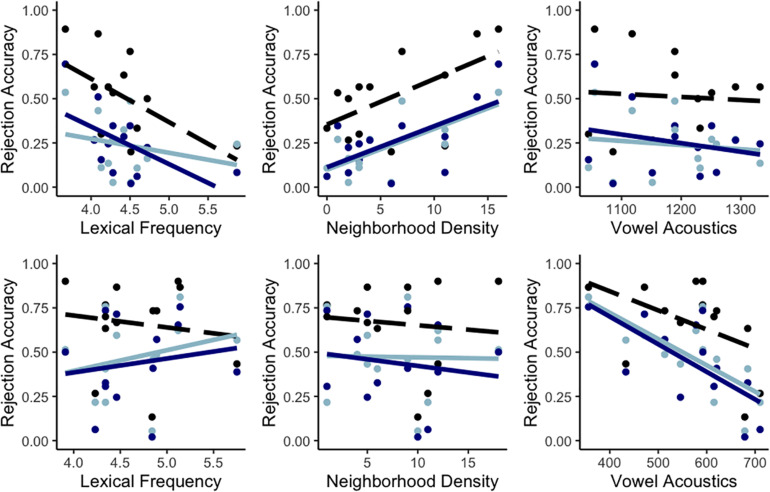
Scatterplots of accuracy in nonword rejection for /æ/-nonwords (top row) and /ε/-nonwords (bottom row) as a function of lexical frequency (left), neighborhood density (center), and vowel acoustics (right) with data split by group. Advanced learners in Llompart (2021) are in black, intermediate learners in Llompart (2021) are in dark blue and intermediate learners in Llompart and Reinisch (2019b) are in light blue. Regression lines (advanced learners in dashed line, intermediate learners in solid lines) are provided for illustration purposes.

Summarizing, model comparisons showed that nonword rejection accuracy for items containing mispronunciations of confusable L2 phones was modulated across the board by both the lexical frequency of the items and the acoustics of the critical vowels. However, the significant interactions and subsequent follow-up analyses indicated that the relative contributions of lexical frequency, neighborhood density and vowel acoustics differed between /æ/-nonwords and /ε/-nonwords. For the former, lower lexical frequencies and higher phonological neighborhood densities contributed to higher accuracies, whereas the F2–F1 values of the critical vowels did not strongly relate to nonword rejection accuracy (see Figure 1, top row). For /ε/-nonwords, higher accuracies in nonword rejection were only associated to lower F2–F1 values (i.e., more [æ]-like) for the critical vowels (see Figure 1, bottom row). Similar scatterplots to those in Figure 1 but with data split by group are provided in Figure 2. An examination of Figure 2 additionally suggests that the asymmetric patterns for the two types of mispronounced nonwords are highly consistent across the three groups of participants included in the sample.

## Discussion

The present study examined the effects of item-specific properties both related to the organization of the L2 lexicon and to the acoustics of the confusable L2 categories on rejection accuracy for nonwords only differing from real words in the phones of a difficult L2 phonological contrast. A series of additional analyses of lexical decision data from German learners of English (Llompart and Reinisch, 2019b; Llompart, 2021) were conducted to assess the effects of i) the lexical frequency of the L2 (non)words presented, ii) their phonological neighborhood densities, and iii) the spectral image of the critical L2 phones, on learners’ ability to reject nonwords containing /ε/-/æ/ mispronunciations. These are factors that have not been considered in previous research but whose thorough investigation could improve our understanding of how lexical properties modulate the phonolexical encoding of phones in challenging L2 contrasts, as well as of the extent to which learners are sensitive to fine phonetic detail regarding the phones in such contrasts when engaging in lexical retrieval tasks. Even though the results of the present study should be interpreted with caution, as they stem from a limited set of L2 (non)words targeting just one L2 contrast and one learner population, they constitute a first stepping stone toward a better characterization of these issues, which are further discussed below.

Before actually gauging the effects of lexical frequency, neighborhood density and vowel acoustics in the present study, however, a first analysis was conducted to assess differences in accuracy as a function of vowel, or item type (/ε/-nonwords vs. /æ/-nonwords), and learner group. This analysis was conducted to confirm previous findings with a larger dataset and, most importantly, so that the model could then be used as a baseline to quantify the effects of the lexical and phonetic predictors of interest at a later stage. Results showed that learners were better at accurately detecting /ε/ → [æ] mispronunciations (/ε/-nonwords; e.g., *l[æ]mon) than /æ/ → [ε] mispronunciations (/æ/-nonwords; e.g., *dr[ε]gon) and that the group of advanced learners included in the analyses outperformed the two groups labeled as intermediate learners. This replicates the findings of previous studies showing nonword rejection asymmetries for words with difficult L2 phonological contrasts (Darcy et al., 2013; Simon et al., 2014; Llompart and Reinisch, 2019b; Melnik and Peperkamp, 2019, 2021) and proficiency and usage effects in nonword rejection for this type of items (Sebastián-Gallés et al., 2005; Amengual, 2016; Llompart, 2021). In addition to this, another relevant finding was that accuracy rates for the two intermediate learner groups, who were recruited and tested at different universities but by means of the same recruiting procedure, were found to be extremely similar. This evidences that the samples from Llompart and Reinisch (2019b) and Llompart (2021) were comparable and speaks in favor of the high reliability of this experimental paradigm when used with late L2 learners and applying systematic recruiting requirements.

The main question was, however, whether lexical frequency, phonological neighborhood density and vowel acoustics influenced nonword rejection accuracy on top of the previously mentioned effects. This was assessed by manipulating the presence or absence of random slopes for the three variables, as well as interaction terms between them and vowel, in the random-effects structure of the models while the fixed-effects structure remained constant. In that respect, results revealed that both the lexical properties of the target items and the acoustics of the critical vowels contributed to characterizing the variation observed for nonword rejection, albeit differently for the two types of items examined. For /æ/-nonwords (i.e., /æ/ → [ε] mispronunciations), lexical factors had robust modulating effects: First, nonwords whose real word counterparts had lower frequencies were more easily rejected than those that had higher frequencies. Secondly, nonwords based on words with more lexical neighbors were more easily rejected than those with fewer neighbors (see Figures 1, 2, top row). In contrast, for /ε/-nonwords (i.e., /ε/ → [æ] mispronunciations), accurate rejection for individual items was tightly related to the acoustics of the critical vowel (/ε/ produced as [æ]), as higher rejection rates were associated to more extremely [æ]-like spectral articulations of /ε/ (see Figures 1, 2, bottom row). Therefore, results showed clear asymmetries between /ε/-nonwords and /æ/-nonwords for the two lexical factors as well as for vowel acoustics.

With regard to vowel acoustics, the fact that it modulated the rejection of /ε/-nonwords (e.g., *l[æ]mon) but not /æ/-nonwords (e.g., *dr[ε]gon) indicates that L2 learners were indeed sensitive to small differences in the acoustic properties of the critical vowels when judging the lexicality of words and similar-sounding nonwords, but only when the mispronunciations in the latter went in one particular direction. A possible explanation for this asymmetry is that the more robust encoding of /ε/ (vs. /æ/) into the lexical representation of L2 words leads not only to higher accuracies when rejecting items in which the vowel is mispronounced, as already shown (Simon et al., 2014; Llompart and Reinisch, 2019b), but also to an enhanced attentiveness to how large (or small) the mismatch between the expected category and the acoustics of the input is. Building on the same argument, the lack of a relationship between vowel acoustics and rejection of *dr[ε]gon-type mispronunciations could be attributed to the “fuzzier” representation of /æ/ in L2 words containing this vowel. This would make L2 learners more tolerant of mispronunciations, and thus less accurate in their judgments, while also reducing their sensitivity to the magnitude of the mismatch between the input and the canonical vowel. In addition, note that, for the L2 contrast of interest, critical differences in peripherality between the two vowels could have also contributed to this asymmetric pattern. Given that /æ/ is more peripheral than /ε/ in the English vowel space, mispronunciations involving a substitution of the less peripheral vowel by the more peripheral one may have been more salient than the opposite type, enhancing the effect that small acoustic differences in the more peripheral region of the vowel space could have on learners’ perception and subsequent decisions (Polka and Bohn, 2003, 2011).

The second asymmetry observed involved lexical frequency and phonological neighborhood density, which were found to only influence rejection accuracy for /æ/-nonwords. For lexical frequency, a potential explanation is that it only played a role for /æ/ → [ε] mispronunciations because these are very often encountered in German-accented English and learners most likely had had experience with items of this kind (Eger and Reinisch, 2019b; Llompart and Reinisch, 2019a, 2020). The effect of lexical frequency could thus be explained by the fact that the more frequent (non)words with /æ/ presented in the task, like *thank*, may have repeatedly been heard as *th[ε]nk in the speech of fellow L1-speakers, while less frequent words like *habit* probably not as much. Consequently, this would have led to learners being more likely to consider *th[ε]nk a real English word than *h[ε]bit. For /ε/-nonwords, on the contrary, as the mispronunciations in these items (/ε/ → [æ]) are not a typical marker of L1-accented speech, the amount of exposure to L1-accented input would not be expected to make a difference, and this would explain the lack of an effect of lexical frequency for these items. Since detailed information about the learners’ L2 input would be needed to be able to properly assess whether L2 input characteristics could indeed be the source of this asymmetry, this explanation remains in need of further research at this point.

Finally, the effect of phonological neighborhood density for /æ/-nonwords indicates that, for the most problematic category in the contrast (i.e., /æ/), the existence of clusters of phonological neighbors containing the same target vowel made it more likely that learners spotted the corresponding mispronunciations^3^. This suggests that high phonological neighborhood densities may support the accurate phonolexical encoding of the vowel into particular L2 lexical representations, probably by strengthening the connection between the challenging non-native phonetic category and the clustered lexical items. For words containing /ε/, phonological neighborhood density may not be as crucial because of the dominant role of /ε/ in the phonological contrast and its relatively easier perceptual identification (Weber and Cutler, 2004; Cutler et al., 2006).

All in all, the present study provides a first approximation to the issue of how the lexicon and speech perception intertwine in the phonolexical encoding of difficult L2 contrasts from an item-centered perspective. Challenging L2 phonological contrasts introduce an additional level of “fuzziness” to L2 lexical representations, which are known to already be fuzzy because of the inherent characteristics of L2 learning itself (Cook and Gor, 2015; Cook et al., 2016; Lancaster and Gor, 2016). Previous studies have shown that, for non-native phonological contrasts in which the two L2 phones differ in how well they match L1 categories, the difficulties brought about by such phones are not symmetric (Weber and Cutler, 2004; Cutler et al., 2006; Darcy et al., 2013; Simonchyk and Darcy, 2017, 2018; Melnik and Peperkamp, 2019, 2021). This study contributes to this literature by suggesting that these asymmetries may also extend to the way in which phonolexical encoding takes place. Based on the present results, the encoding of the best-fitting or dominant L2 category (i.e., /ε/) appears not to be strongly constrained by lexical properties of specific L2 lexical items such as lexical frequency and phonological neighborhood density. This, in addition to the effects of vowel acoustics observed for /ε/-nonwords, suggests that, for this category, encoding may be more directly linked to learners’ phonetic perception of the contrast. Note that this idea accounts well for the results of Llompart and Reinisch (2019b), who found that it was only for responses to (non)words with phonological /ε/ (and not /æ/) that a relationship with learners’ perceptual flexibility in a distributional learning task could be found. In contrast, for the worse fitting, non-dominant category (i.e., /æ/), lexical decision data suggests that the level of success at phonologically encoding the non-native phonetic category into lexical representations is influenced by higher-level lexical properties that situate these items within the learners’ vocabulary, and possibly relates to their familiarity with native and non-native input. Hence, the phonolexical encoding of /æ/ could be hypothesized to operate to a larger extent in a piecemeal manner (Lieven et al., 1997; Pine and Lieven, 1997) modulated by the learners’ experience with the L2 and even with particular L2 words (Llompart, 2019, 2021). Future research including larger item samples and, ideally, also examining data from other experimental paradigms that tap into lexical retrieval will now be essential to ascertain to what extent the insights gained from nonword rejection in this study are robust and generalizable.

## Data Availability Statement

Publicly available datasets were analyzed in this study. This data can be found here: https://osf.io/u7syd/ (Open Science Framework).

## Ethics Statement

Ethical review and approval was not required for the study on human participants in accordance with the local legislation and institutional requirements. The patients/participants provided their written informed consent to participate in this study.

## Author Contributions

ML was solely responsible for the conception of the study, the analysis and interpretation of the data, and the drafting of the manuscript.

## Conflict of Interest

The author declares that the research was conducted in the absence of any commercial or financial relationships that could be construed as a potential conflict of interest.

## Footnotes

1 To illustrate the ceiling effects for real word acceptance, percentages of correct responses to real word stimuli by vowel (/ε/-words and /æ/-words) for the different groups tested in Llompart and Reinisch (2019b) and Llompart (2021) are provided as Supplementary Table 1. In addition, Supplementary Table 2 outlines the results of a model with the exact same structure as the base model reported in the “Results” section but run on responses to real words instead of mispronounced nonwords. Unlike the model in the Results section, the model for real words does not show significant differences in accuracy as a function of vowel or learner group.

2 The elicited measures of language use differed across studies, and are thus not fully comparable. By contrast, proficiency measures for English comprehension and spoken English were the same for the two studies, albeit elicited using slightly different scales. In spite of this, when the means for all three groups of participants as reported in the original studies are transformed into a common 0–10 scale (0 = very poor; 10 = very good), it is observable that the intermediate learners in Llompart and Reinisch (2019b) and Llompart (2021) provided very similar scores for both English comprehension (2019: 6.74; 2021: 6.5) and spoken English (2019: 5.5; 2021: 5.88), while the advanced learners in Llompart (2021) self-scored their abilities as higher for both measures (English comprehension: 7.55; spoken English: 6.72).

3 As an additional check for phonological neighborhood density, it was assessed whether densities per item differed from these reported in Table 1 when only neighbors containing the same target vowel were considered (vs. all neighbors). A correlational analysis showed that total neighborhood densities and neighborhood densities only including same-vowel neighbors were almost perfectly correlated [*r*(24) = 0.98, *p* < 0.001].
