# Supplementary material for: Lexical and Phonetic Influences on the Phonolexical Encoding of Difficult Second-Language Contrasts: Insights From Nonword Rejection
Source: Front Psychol. 2021 May 31;12:659852. doi: 10.3389/fpsyg.2021.659852 (PMC8200638; doi:10.3389/fpsyg.2021.659852)
Supplement: Supplementary file 1 [file Table_1.DOCX]

Supplementary Material

Table S1*.* Percentages of correct responses to real word stimuli by vowel (/ɛ/-words and /æ/-words) for the learner groups tested in Llompart and Reinisch (2019b) and Llompart (2021). Standard deviations are provided in parentheses.

| Group of learners | Real /ɛ/-words | Real /æ/-words |
| --- | --- | --- |
| Intermediate learners:  Llompart and Reinisch (2019b) | 96.88% (17.42) | 96.67% (17.95) |
| Intermediate learners:  Llompart (2021) | 97.63% (15.23) | 95.6% (20.52) |
| Advanced learners:  Llompart (2021) | 97.95% (14.19) | 96.92% (17.29) |

Table S2*.* Coefficients and significance of values for a generalized linear mixed effects model assessing the effects of Vowel (/ɛ/-/æ/), Proficiency (Intermediate / Advanced), Study (Llompart & Reinisch, 2019b / Llompart, 2021) and the interactions between Vowel and Proficiency and Vowel and Study on response accuracy (0 = incorrect / 1 = correct) for real words containing /ɛ/ and /æ/ in Llompart and Reinisch (2019b) and Llompart (2021). The structure of the model mirrors that of the base model reported in the Results section for responses to mispronounced nonwords with /ɛ/ and /æ/ in the same studies.

| Predictor | *b* | *z* | *p* |
| --- | --- | --- | --- |
| Intercept | 3.55 | 21.84 | < .001 |
| Vowel | -0.42 | -1.49 | .14 |
| Proficiency | 0.36 | 0.97 | .33 |
| Study | -0.003 | -0.01 | .99 |
| Vowel x Proficiency | -0.10 | -0.14 | .89 |
| Vowel x Study | -0.57 | -1.17 | .24 |
